# Keratin-Laden Bioink for Corneal Stroma Bioprinting

**DOI:** 10.3390/bioengineering13060670

**Published:** 2026-06-09

**Authors:** Leon Balters, Stephan Reichl

**Affiliations:** Institute of Pharmaceutical Technology and Biopharmaceutics, Technische Universität Braunschweig, 38106 Braunschweig, Germany; l.balters@tu-braunschweig.de

**Keywords:** bioprinting, cornea, keratin, hyaluronic acid, collagen, keratocytes, corneal fibroblasts

## Abstract

Corneal blindness remains a major clinical challenge, yet donor grafts are scarce. Bioprinting has emerged in recent decades to potentially overcome donor shortage. In bioprinting, collagen is a common biomaterial. However, one alternative biomaterial, which has shown promising results in corneal tissue engineering, is keratin. Therefore, human hair keratin was investigated in this study as a bioink component for stroma bioprinting. Two keratin preparations, an aqueous extraction and an alkaline extraction, were incorporated into a methacrylated hyaluronic acid bioink and compared with a collagen methacrylated hyaluronic acid bioink. Corneal stroma-like constructs were printed by extrusion bioprinting and evaluated for optical transmission, biomechanical properties, cell compatibility and protein expression of collagen type I and alpha-smooth muscle actin over a four-week period. Two different cell types, immortalized corneal keratocytes and human corneal fibroblasts, were used. The alkaline keratin dialysate-supplemented bioink showed similar optical transparency and biomechanical properties to the collagen-supplemented bioink. Cell viability was high in all formulations. Protein expression of collagen type I and α-smooth muscle actin remained low in all bioinks. Human corneal fibroblasts appeared to be in a quiescent state and were unable to produce large amounts of collagen. This comparative study between collagen and keratin is a first step towards establishing keratins in bioprinting, leading to more complex bioinks.

## 1. Introduction

Vision is one of the most vital senses of a human being, enabled largely by the cornea, which provides roughly two-thirds of the eye’s refractive power [1]. Especially the stroma contributes the most to refraction as it makes up the largest part of the cornea [2]. The stroma is composed of highly organized collagen fibrils interspersed with specialized keratocytes [2]. Conditions such as keratoconus, dystrophies or trauma-induced scarring can disrupt stromal organization, leading to corneal opacity and, in severe cases, to corneal blindness [3]. When medical treatment fails, a full or partial corneal transplantation remains the clinical gold standard for restoring vision [4]. Yet, the availability of donor corneas is limited, and allografts carry a risk of rejection or immune response [5,6]. This has sparked interest in corneal tissue engineering [7]. Emerging technologies aim to produce substitutes that overcome donor shortages.

One area of tissue engineering is bioprinting. Bioprinting is an additive-manufacturing process that involves, in most cases, cells and a hydrogel. Extrusion-based bioprinting is one of the most used bioprinting techniques as it is affordable and easy to use [8]. Unlike traditional manual casting, bioprinting offers advantages such as superior spatial control, automation and reproducibility, while allowing for the precise customization of graft geometry and thickness [9,10,11,12]. Corneal bioprinting is a highly complex field, as the hydrogel must not only provide a suitable microenvironment for cells, but must also be transparent and have mechanical properties to withstand the intraocular pressure [13]. In the past, many different materials have been tested, including natural biomaterials, such as gelatin, alginate or collagen, and synthetic materials, such as poly (ethylene-glycol) diacrylate (PEGDA) [9]. In order to utilize the advantages of natural and synthetic materials, biomaterials are often chemically modified, for example, gelatin methacryloyl.

Hyaluronic acid (HA), a naturally occurring glycosaminoglycan, can be found in abundance in human tissue [14]. It is an attractive biomaterial for bioprinting as it has proven biocompatibility and also the possibility for chemical modification [15]. To our knowledge, methacrylated hyaluronic acid (HAMA) has not been widely applied in the corneal bioprinting field [9,16]. With the addition of methacrylation groups, it can be photo-crosslinked to yield a stable hydrogel. HA can be found in the cornea as a regulator for wound healing after injury [17,18]. However, HA or HAMA lacks cell-adhesive motifs, so supplementation or modification with a protein or peptide sequence is desirable to promote cell attachment and physiological behavior.

To incorporate proteins, collagen type I is a commonly used protein. Collagen type I is the most abundant protein in the corneal stroma and, thus, has been widely used in corneal bioprinting [9]. However, collagen alone lacks mechanical stability, and, therefore, it is often combined with other biomaterials [19]. In addition, collagen is usually obtained from animals, which is ethically controversial, or produced through recombinant engineering, which makes it relatively expensive.

An alternative to collagen could be keratins. Keratins are a family of structural proteins found in hair or nails. Keratins can be gained from human hair, which is a waste product and often not further used, and have been applied in tissue engineering [20,21,22]. Similar to collagen, keratins show cellular attachment sites such as arginine–glycine–aspartic acid (RGD) and leucine–aspartic acid–valine (LDV), making them suitable for tissue engineering and regenerative medicine [21]. For example, in wound healing, keratin-based materials have demonstrated biocompatibility and hemostatic effects [23]. Furthermore, keratin-based materials have been studied for ocular surface applications [24,25,26]. Keratin films showed similar epithelial wound healing to amniotic membranes [27]. In addition, in an in vivo rabbit model, keratin films demonstrated better epithelial wound healing than amniotic membranes [28]. In another in vivo study, keratin films demonstrated good biocompatibility and transparency [29]. These previous findings support further use of keratins in ocular surface applications and, thus, enable them for use in bioprinting as their potential remains largely unexplored.

To overcome keratin insolubility and isolate the protein from hair, the Shindai method was applied here. In this study, two keratin preparations, an aqueous keratin dialysate and an alkaline keratin dialysate, were compared with collagen in a HAMA bioink [24]. Aqueous keratin dialysate becomes a suspension of keratin nanoparticles because of the occurrence of oxidative disulfide reformation. In contrast with aqueous keratin dialysate, alkaline dialysate is a clear solution. Another difference is the average molecular weight, which is lower in alkaline dialysate. In addition, the content of free thiol groups is higher in alkaline dialysate [24].

For a proof of concept, discs similar in size to corneal stroma were printed and characterized for optical transparency, biomechanical properties and biocompatibility. For biocompatibility, two different cell types were used: an immortalized human corneal keratocyte cell line and primary human fibroblasts isolated from human corneas. Corneal keratocytes represent the healthy state of the cornea, while fibroblasts are more active and are involved in corneal wound healing [30]. Different cells were used to see how they interact with the artificial 3D bioprinted matrix. Typical cell marker expression, such as collagen type I and α-smooth muscle actin (αSMA), was monitored over four weeks. Our work aims to expand the choice of biomaterials for corneal bioprinting. Keratins as an alternative or similar biomaterial to collagen could represent a cost-effective and ethically safe alternative. This study lays the foundation for further studies on keratins in the field of bioprinting.

## 2. Materials and Methods

### 2.1. Materials

Hyaluronic acid was purchased from Acros Organics, Geel, Belgium. Dimethylformamid was obtained from VWR Chemicals, Radnor, PA, USA. Methacrylic anhydride was purchased from Alfa Aesar, Haverhill, MA, USA. Sodium hydroxide, sodium chloride, sodium dodecyl sulfate (SDS), urea, thiourea, mercaptoethanol, sodium bicarbonate, calcium chloride, ascorbic acid, paraformaldehyde and Triton-X-100 were obtained from Carl Roth, Karlsruhe, Germany. A SpectraPor^®^ dialysis membrane (MWCO 6–8 kDa) was purchased from Repligen, Waltham, MA, USA. Vivaspin^®^ 20 (MWCO 5 kDa) was acquired from Sartorius, Göttingen, Germany. TRIS was obtained from Caesar & Loretz, Hilden, Germany. Deuterium hydroxide was obtained from Sigma-Aldrich, St. Louis, MO, USA. Sucrose, fetal bovine serum (FBS), antibiotic/antimycotic solution, dispase, insulin, polysorbate 20 (Tween^®^ 20) and bovine serum albumin (BSA) were purchased from Sigma-Aldrich, München, Germany. Keratinocyte growth medium (KGM) was obtained from Lonza, Basel, Switzerland. DMEM and L-glutamine were purchased from Biochrom, Berlin, Germany. Phosphate-buffered solution (PBS) was obtained from MP Biomedicals, Irvin, CA, USA. Collagenase was purchased from Merck Millipore, Darmstadt, Germany. TGF-ß1 was obtained from PeproTech, Hamburg, Germany. Lithium phenyl (2,4,6-trimethylbenzoylphosphinat) (LAP) was obtained from BLDPharm, Shanghai, China. Normal goat serum was purchased from Vector Lab, Peterborough, UK. Rabbit anti-collagen antibody, mouse anti-αSMA, FITC-conjugated anti-rabbit antibody and AlexaFluor568-conjugated anti-mouse antibody were obtained from Abcam, Cambridge, UK. Hoechst33342 was purchased from Thermo Fisher Scientific, Waltham, MA, USA. Collagen type I was extracted from rat tails using an internal standard procedure.

### 2.2. Synthesis of HAMA

Methacrylated hyaluronic acid (HAMA) was prepared according to earlier reports [31]. Briefly, high molecular HA (2000 kDa) was dissolved in double-distilled water and stirred overnight at room temperature. On the next day, dimethylformamide was added in a 1:2 DMF/water ratio. Methacrylic anhydride was added dropwise, while maintaining the pH between 8 and 9 with NaOH. After 20 h of stirring in the dark, the reaction was neutralized, diluted with water and adjusted with sodium chloride (0.6 M). The solution was then transferred to a dialysis membrane (SpectraPor^®^ MWCO 6–8 kDa) and dialyzed against deionized water at 4 °C for 10 days, after which it was lyophilized and stored at −20 °C until further use. The successful methacrylation was monitored by ^1^H-NMR using a Bruker AV III HD 500 Spectrometer (Billerica, MA, USA). Samples were prepared in D_2_O and measured at room temperature, using tetramethylsilane as an internal standard.

### 2.3. Keratin Extraction

Human hair was collected from multiple donors at a local hairdresser. The hair was pooled and washed in 0.5% SDS, rinsed thoroughly with water and air-dried. Keratin was then extracted following the Shindai protocol [32]. To prepare the Shindai solution, 25 mM TRIS, 2.6 mM thiourea, 5 M urea and 5% 2-mercaptoethanol were dissolved in water and the pH was adjusted to 8.5. An amount of 20 g of hair was stirred gently in 400 mL of Shindai solution for 72 h at 50 °C. Subsequently, the mixture was centrifuged at 5000 rpm for 30 min, and the supernatant (Shindai extract) was filtered and stored at −20 °C until further use.

To remove cytotoxic agents such as thiourea and 2-mercaptoethanol, the extract underwent extensive dialysis against water or 0.25 M NaOH. The extracted keratin was intensively analyzed in previous studies [24,26,33]. For the aqueous dialysis, the extract was dialyzed against water in a 1:50 ratio (*v*/*v*) for 6 days at room temperature using an MWCO 6–8 kDa cellulose dialysis tube (SpectraPor^®^) with a daily change of water. The dialyzed solution was then centrifuged for 30 min at 10,000 rpm for purification. For alkaline dialysis, the extract was dialyzed against 0.25 M NaOH in a 1:50 ratio (*v*/*v*) for 6 days at 4 °C with a daily change of medium. The alkaline fraction was concentrated using 5 kDa Vivaspin^®^ 20 and then repeatedly diluted with 0.05 µM NaOH.

### 2.4. Cell Culture

#### 2.4.1. Human Corneal Keratocytes (HCKs)

SV40 immortalized human corneal keratocytes (HCKs), kindly provided by Dr. M. Zorn-Kruppa, were used [34]. The HCKs were cultured in KGM supplemented with 0.5 M CaCl_2_ under standard conditions (37 °C and 5% CO_2_) in a humidified atmosphere.

#### 2.4.2. Human Corneal Fibroblasts (HuFibs)

Human primary corneal stromal cells were isolated from transplant-discarded donor corneas provided by Gesellschaft für Transplantationsmedizin Mecklenburg-Vorpommern gGmbH (Rostock, Germany). For enzymatic isolation, the stroma was first separated from the sclera and epithelium using a scalpel, rinsed in PBS, and then digested in dispase solution (1.5 U/mL) at 37 °C for 1 h. After a second PBS wash, the tissue was further digested in 100 U/mL of collagenase for 12 h on a shaker. The resulting cell suspension was centrifuged at 1500 rpm for 10 min, and the pellet was resuspended in DMEM containing 10% FBS, 4 mM L-glutamine and a 1% antibiotic/antimycotic solution and cultured at 37 °C and 5% CO_2_. To even out donor-to-donor variability, cells from five different corneas were pooled. Cell passages of 3–5 were used for bioprinting. Post-printing, constructs were kept in a differentiation medium (DMEM, 10% FBS, 4 mM L-glutamine, 1% antibiotic/antimycotic, 10 µg/mL insulin, 50 µg/mL vitamin C, and 1 ng/mL TGF-ß1) to enhance cell proliferation and collagen production [35].

### 2.5. Bioprinting

Based on a previous study, a concentration of 30 mg/mL HAMA and 1.32 mM photoinitiator LAP was selected. Shape fidelity and printability was previously demonstrated [36]. Aqueous or alkaline keratin dialysates were added in a 0.7:1.8 (*v*/*v*) ratio to the bioink. Prior to using alkaline keratin dialysate, the solution was adjusted to pH 7.4 using acetic acid and 10× MEM, L-glutamine (12.9 mM) and NaHCO_3_ (16.1 mg/mL). For the HAMA collagen bioink, type I collagen was added to reach 1.344 mg/mL. Collagen was neutralized using 10× MEM, L-glutamine (12.9 mM) and NaHCO_3_ (16.1 mg/mL). HCKs or HuFibs were suspended in the bioink at a concentration of 1.5·10^6^/mL. Four different bioinks were created: HAMA, HAMA with aqueous keratin dialysate (HAMAKerW), HAMA with alkaline keratin dialysate (HAMAKerAlk) and HAMA with collagen type I (HAMACol).

Bioprinting was carried out on a BioScaffolder 3.3 (GeSiM mbH, Radeberg, Germany) with pneumatic extrusion. To evaluate the hydrogel, a disc modeled after the cornea was printed. It consisted of three layers with a total thickness of 0.5 to 0.6 mm and a diameter of 11 mm. The samples were printed using pneumatic extrusion using a 250 µm nozzle, a printing speed of 5 mm/s, and a pressure range of 50–60 kPa. Post-printing, the samples were crosslinked using a UV solo pen of 405 nm (Opsytec Dr. Gröbel GmbH, Ettlingen, Germany). The UV pen was positioned at a distance of 50 mm for 6 s, providing an estimated irradiance of 100 mW/cm^2^ based on the manufacturer’s specifications.

### 2.6. Cell Viability (Live/Dead Staining)

Cell viability was directly visualized by staining with 5 µM of calceinAM and 2 µM of ethidium homodimer I. After incubation for 30 min, the samples were imaged under a fluorescence microscope (Olympus IX50 with DLP28, Olympus, Tokyo, Japan). Live/dead assays were carried out at 2 h post-printing (=day 0) and again on days 7, 14 and 28.

Furthermore, a relative fluorescence index was calculated. Fluorescence images were analyzed using ImageJ software (version 1.53e), and the fluorescence area for calceinAM and ethidium homodimer I was measured. The relative fluorescence index was calculated according to the following equation:

$$relative\;fluorescence\;index\;\left(\%\right)=\;\frac{Area\;of\;calceinAM}{\left(Area\;of\;calceinAM\;+\;Area\;of\;ethidium\;homodimer\;I\right)}\;\times 100$$

Analysis was performed on three independent images for each sample and timepoint.

### 2.7. Light Transmission

Extinction was measured in the wavelength range of 300–800 nm at 1 nm intervals using an Infinite M Plex spectrophotometer (Tecan, Männedorf, Switzerland), and the corresponding light transmission was calculated. Before measurement, each construct was rinsed with PBS. The three-dimensional-printed constructs were kept moist and were placed in the middle of a 6-well plate for measurement. To assess macroscopic clarity, each disc was also placed over a printed “B”. Measurement was conducted at 2 h post-printing (=day 0) and again on days 7, 14 and 28.

The refractive index was measured at 25 °C on an Abbemat-WR-refractometer (Anton Paar, Ostfildern-Scharnhausen, Germany).

### 2.8. Mechanical Testing

Biomechanical properties were evaluated on Zwicki-Line Z 0.5 machine (Zwick, Ulm, Germany) with a 10 N load cell, and the testXpert^®^ II software (version 3.0) was used for analysis. For compression testing, hydrated discs were preloaded to 0.03 N and then compressed at a rate of 1 mm/min. Compression tests were carried out 2 h post-printing (=day 0) and on days 7, 14 and 28. For tensile testing, a modified version of ASTM D412c was printed [37]. After applying a 5 mN preload at 10 mm/min, Young’s modulus was recorded at 5 mm/min and until sample failure. The test speed was set to 25 mm/min.

### 2.9. Swelling

Disc diameter was measured with the Zeiss Labscope software (version 4.3.2) based on images taken with a stereomicroscope and camera (Stemi 508 and Axiocam208, both Zeiss, Jena, Germany). Measurements were made immediately after printing and again after 2 h (=day 0) and 7, 14 and 28 days. Size increases were calculated as percentage changes.

### 2.10. Indirect Immunofluorescence (IF)

At days 7, 14 and 28 after printing, 3D-printed discs were fixed for IF by incubating them in 4% paraformaldehyde and 10% sucrose in PBS for 1 h, followed by thorough washing with PBS. The discs were permeabilized in 0.1% Triton-X-100 for 30 min and then rinsed again with PBS three times. To block nonspecific sites, the samples were incubated with 10% normal goat serum and 0.1% Tween^®^ 20 in PBS for 1 h. The blocking solution was then removed and replaced with the antibody solution, consisting of 1:175 rabbit anti-collagen I and 1:200 mouse anti-αSMA in a TRIS buffer containing Triton-X-100 and BSA. After 48 h of incubation, the primary antibody solution was removed, the discs were washed three times with PBS and secondary antibodies were applied for 24 h. Finally, the discs were washed again with PBS, nuclei were stained with Hoechst 33342 and imaging was performed on an Olympus IX50 fluorescence microscope (with Olympus DLP28). The acquired images were analyzed using Olympus cellSense software (version 4.2.1).

### 2.11. Statistical Analysis

Unless otherwise stated, all measurements were carried out at least three times. The data are presented as means ± standard deviation. The data were analyzed with one-way analysis of variance (ANOVA) with a Tukey post hoc test using the OriginLab 2024b software. Prior to ANOVA, the data were tested for outliers using the Grubbs’ test and for a normal distribution using the Shapiro–Wilk test, and homoscedasticity was assessed using Levene’s test. If homoscedasticity was significant with Levene’s test, a Welch ANOVA was conducted. A significance level of * *p* < 0.05 was used and was considered statistically significant.

## 3. Results and Discussion

### 3.1. Bioprinting

**Figure 1:**
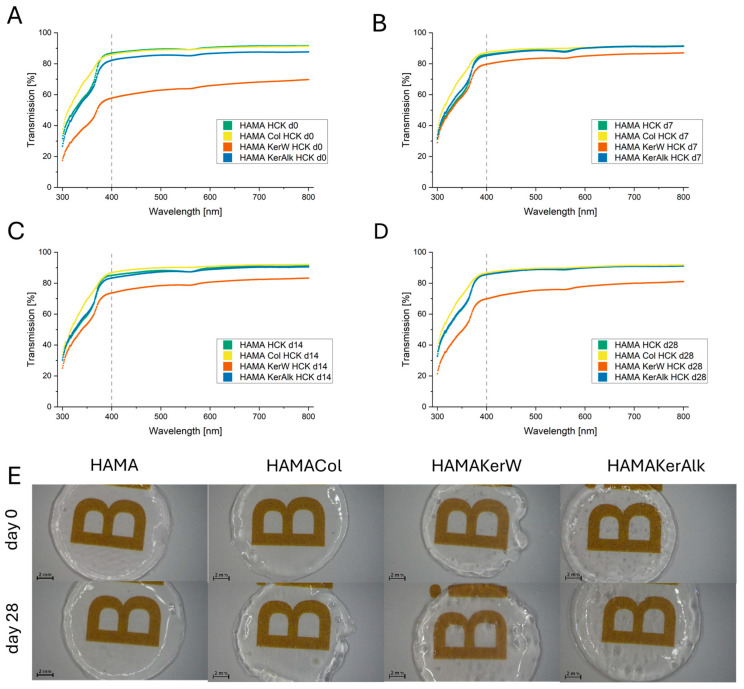
Light transmission on day 0 (**A**), day 7 (**B**), day 14 (**C**) and day 28 (**D**) of HAMA, HAMACol, HAMAKerW and HAMAKerAlk across 300–400 nm and the visible spectrum (400–800 nm). Macroscopic images of bioprinted discs on day 0 and day 28 (**E**) of HAMA, HAMACol, HAMAKerW and HAMAKerAlk.

A previous study investigated the rheological properties and printability of pure HAMA bioink [36]. In the current study, the functional printability and shear-thinning behavior were maintained even after the addition of keratin or collagen, as evidenced by the high morphological similarity and uniformity across all bioink formulations (Figure 1E).

#### 3.1.1. Light Transmission

Transparency is crucial when engineering corneal stromal tissue. Therefore, light transmission of hydrogel discs containing HCKs across 300–400 nm and the visible spectrum (400–800 nm) was measured. HAMA and HAMA hydrogels with the addition of collagen or alkaline keratin dialysate maintained very high transparency of over 80% throughout the entire period of four weeks (Figure 1A–D). Adding aqueous keratin dialysate initially reduced transmission to around 60% on the first day (Figure 1A). This might have been due to the nanoparticles of aqueous keratin dialysate. It then rose over the first two weeks to nearly 80% before slightly declining again by week 4. A decrease over time could have been due to a high cell concentration, as the formation of dense cells scatters the light and thus decreases the transmission [38]. However, more cells were visible in the HAMACol than in the HAMAKerW bioink (Figure 3). Furthermore, more viable cells could be seen in the HAMACol bioink than in the HAMAKerAlk bioink, whereas both demonstrated high transmission on day 28 (Figure 1D). Therefore, a higher cell concentration was probably not a plausible explanation for the change in light transmission, and the difference in the HAMAKerW bioink was more likely due to the nanoparticles. Nonetheless, all printed discs remained sufficiently clear so that the letter “B” was still readable (Figure 1E). Macroscopically, cell clusters could be seen in the HAMACol and HAMAKerW hydrogel discs.

Furthermore, the refractive index of the hydrogels was determined. As expected for hydrogels with a high water content, the measured values were close to water. The refractive index of the cornea is reported to be between 1.335 and 1.4391 [39]. The refractive indices were 1.3353 ± 0.0002 for HAMA, 1.3415 ± 0.0002 for HAMACol, 1.3360 ± 0.0004 for HAMAKerW and 1.3361 ± 0.0004 for HAMAKerAlk. Although all four formulations were at the lower end of the reported values, the collagen-containing bioink showed a significantly higher index. Nevertheless, the addition of aqueous or alkaline keratin dialysate to HAMA increased the refractive index slightly. However, the addition of aqueous keratin dialysate to the HAMA gel appeared to be inferior to the more common biomaterial collagen and alkaline keratin dialysate in terms of refractive index and transmission.

#### 3.1.2. Mechanical Testing and Swelling

**Figure 2:**
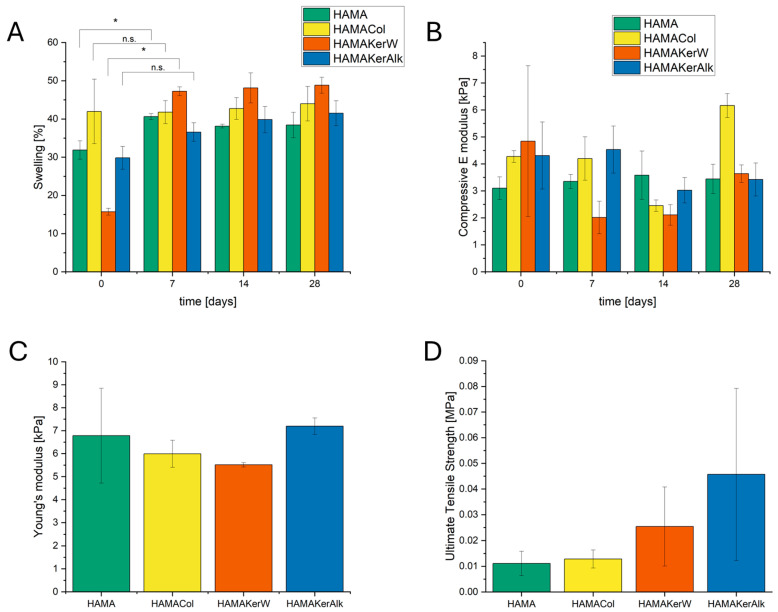
(**A**) Swelling of the HAMA, HAMACol, HAMAKerW and HAMAKerAlk discs over the course of 28 days. (**B**) Compressive elastic modulus of the HAMA, HAMACol, HAMAKerW and HAMAKerAlk discs over the course of 28 days. (**C**) Young’s modulus of HAMA, HAMACol, HAMAKerW and HAMAKerAlk discs by tensile testing. (**D**) Ultimate tensile strength of HAMA, HAMACol, HAMAKerW and HAMAKerAlk. (* = significance level of *p* < 0.05; n.s. = non-significant).

Hydrogel swelling, which reflects hydrophilicity, is considered to be another relevant parameter for corneal application [40]. Therefore, the discs’ swelling behavior over four weeks was tracked. The HAMACol and HAMAKerAlk reached an equilibrium on the first day, showing no significant change through day 7, whereas the HAMA and HAMAKerW stabilized after day 7 and remained unchanged for the next three weeks (Figure 2A). A predictable, limited degree of swelling may be advantageous in engineered corneal constructs, as this might mimic natural hydration and supports cell viability as long as optical clarity is preserved [41]. Uncontrolled swelling in corneal grafts can signal failure and impair transparency [42,43]. Therefore, a rapid equilibrium is desirable, which is why the HAMACol and HAMAKerAlk outperformed HAMA and HAMAKerW. However, it should be noted that further data points between 2 h and 7 days are required to more precisely determine when equilibrium is reached and to fully understand the factors contributing to these differences.

Mechanical behavior was evaluated by two approaches: a compression test on the bioprinted constructs over 28 days and a tensile test on a dumbbell specimen using a modified ASTM D412c printed construct. The compressive elastic modulus of the discs remained unchanged over the four weeks except for HAMACol on day 28, where a high increase in elastic modulus could be seen (Figure 2B). Nevertheless, the discs showed elastic moduli in the range of around 2–6 kPa, which reflects a highly elastic material. Tensile testing produced a similar Young’s modulus of about 6 kPa (Figure 2C). However, a trend could be seen where the ultimate tensile strength (UTS) as well as the strain at breakage (Figure S5) increased with the additions of aqueous and alkaline keratin dialysate (Figure 2D). All formulations show strain-hardening behavior, seen by an increase in the slope (Figure S5). No significant differences could be seen in the measurements of the UTS and Young’s modulus. This could be attributed to either the addition of those proteins being too small or HAMA itself being too dominant to allow for a change in behavior. Although these findings fail at the very lower end of reported corneal values, the printed discs were still easily handled with tweezers [44]. To further verify whether the discs have sufficient mechanical strength, additional mechanical tests such as suture tests and ex vivo or in vivo application are recommended.

#### 3.1.3. Cell Viability

Cell viability serves as a primary indicator of biocompatibility. It is critical in tissue engineering, especially in bioprinting, to maintain high survival. Both the bioink composition and the processing steps, such as crosslinking and extrusion printing, can influence cell viability. In particular, extrusion-based printing imposes greater mechanical stress and, thus, has a larger impact on viability compared with other bioprinting methods [8]. It is, therefore, highly valuable to check the viability shortly after printing to evaluate the initial printing process.

**Figure 3:**
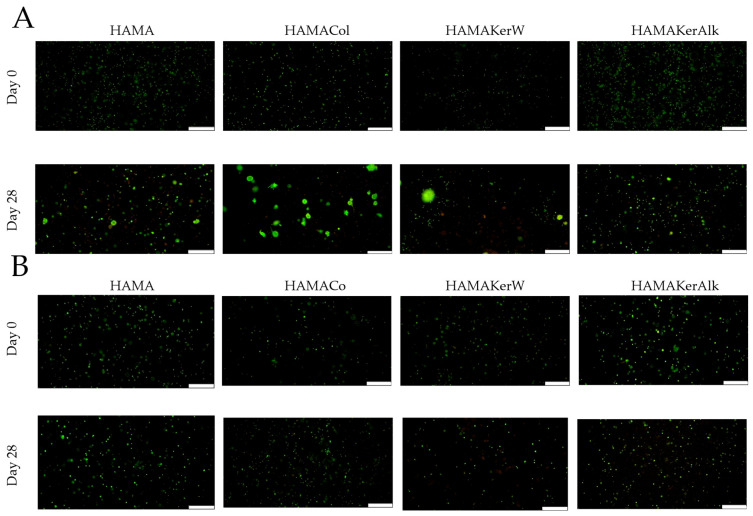
(**A**) Live/dead staining of HCKs in HAMA, HAMACol, HAMAKerW and HAMAKerAlk on day 0 and on day 28; scale bar is equal to 500 µm. (**B**) Live/dead staining of HuFibs in HAMA, HAMACol, HAMAKerW and HAMAKerAlk on day 0 and on day 28; scale bar is equal to 500 µm. Note for (**B**) HAMACol on day 14 is depicted; live/dead staining on days 7 and 14 can be found in the supplementary data in Figures S1 and S2.

The cell viability of HCKs and HuFibs, encapsulated within the HAMA, HAMACol, HAMAKerW and HAMAKerAlk bioinks, was monitored over 28 days using live/dead staining at days 0, 7, 14 and 28. On day 0, both cell types showed very high survival and relative fluorescence indices in all four hydrogels, confirming they tolerated the extrusion and UV-crosslinking steps (Figure 3A and Figure S3A). HCKs remained viable at all time points, indicating that neither gel is cytotoxic. However, the viability and the relative fluorescence index in HAMAKerW decreased over the course of 28 days (Figure S3A). In the HAMACol bioink, formation of cell clusters could be clearly seen on day 28, indicating an increase in cell number and, thus, a likely faster proliferation compared with the other bioinks. Cell clusters could be seen to a lesser extent in HAMA, HAMAKerW and in HAMAKerAlk. This suggests that collagen might send stronger cues that lead to an increase in cell number than the addition of keratins or without. Since this is merely a qualitative analysis that provides preliminary data, further research is required for the bioinks in the future, especially additional quantitative analyses of the proliferation rate, such as the Alamar Blue assay.

HuFibs likewise maintained high viability and relative fluorescence indices throughout the time period (Figure 3B and Figure S3B) but showed no changes in morphology, implying they stayed alive in a more quiescent state. Even though HCKs showed an increase in cell number in the HAMACol bioink, it could not be seen with the HuFibs. A slight decrease in viability and the relative fluorescence index was also visible in the HAMAKerW and HAMA bioink (Figure S3B). This four-week-period data suggest that the addition of aqueous keratin has a marginally lower impact on cell viability than the other bioinks. However, further research is needed to confirm this trend, especially with another quantitative analysis, such as an MTT cell viability assay.

Although both cell types exhibited high viability, they differed in cell number and morphology. While HCKs are typically quiescent in vivo, their immortalization may have overridden this function and led to a more proliferative type that can also be observed in 2D culture. Nevertheless, the typical dendritic shape seen in 2D and other 3D culture models of HCKs could not be seen here [34,45]. Meanwhile, HuFibs remained in a quiescent state and showed no sign of proliferation, even though they were cultured in a differentiation medium. This suggests that the 3D-bioprinted environment for the HuFibs might have diminished the cues sent by the differentiation medium. Therefore, this 3D-bioprinted environment could be used to study the behavior of HuFibs in their quiescent, native state.

#### 3.1.4. Indirect Immunofluorescence (IF)

To evaluate the expression of collagen type I and αSMA in both cell types, HCKs and HuFibs, IF was conducted at days 7, 14 and 28.

Collagen type I is the predominant protein in the cornea and is produced at high levels by both HCKs and HuFibs in vitro [35,46]. Collagen fibers form the highly ordered fibrillar network of the corneal stroma [47]. In contrast, αSMA is expressed by myofibroblasts. Myofibroblasts emerge after in vivo injury and are responsible for the structure and reorganization of the extracellular matrix [48,49,50]. In 2D culture, HuFibs can express αSMA when the medium is supplemented with TGF-ß1. Since the medium used here was supplemented with TGF-ß1, a higher expression of αSMA was expected [35]. Meanwhile, HCKs should not express αSMA under the used conditions here [46].

**Figure 4:**
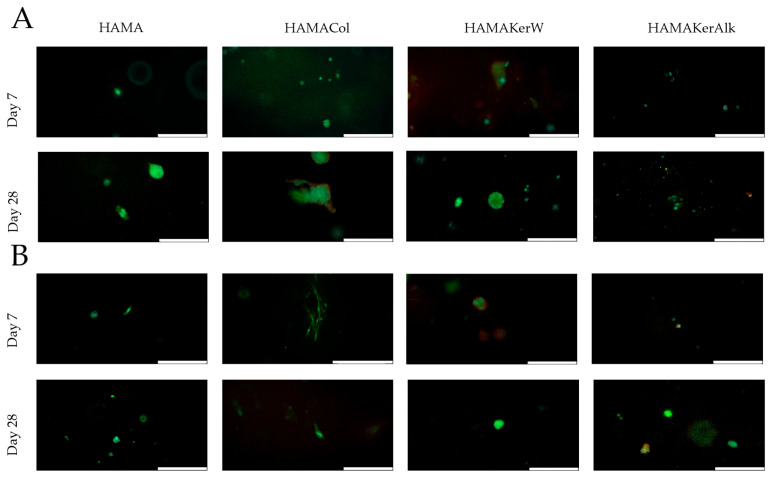
(**A**) IF of HCKs, green fluorescence is collagen type I, red fluorescence is αSMA and blue is hoechst33342. (**B**) IF of HuFibs; scale bar is equal to 200 µm except HAMAKerW on day 0, scale bar is equal to 100 µm. (**B**) HAMACol on day 0; scale bar is equal to 500 µm. Note: Non-merged IF staining as well as day 14 can be seen in the supplementary data in Figures S4 and S5.

On day 7, HCKs in all four bioinks produced only trace amounts of collagen type I (Figure 4A). In HAMAKerW, starting on day 7, αSMA was expressed, while in HAMACol and HAMAKerAlk, very small amounts were determined. Over the next three weeks (through day 28), levels of both proteins rose in all formulations, but collagen I was consistently higher in the collagen-containing bioink, mirroring the greater cell number seen in the live/dead assays. However, in the HAMAKerAlk, only traces of collagen type I could be seen on day 28. A study conducted by Choi et al. showed a decrease in collagen type I of HuFibs in a pure collagen bioink [51]. However, the medium used in their study differs from the medium used in this study. The results presented here could indicate that incorporating collagen improves matrix protein production compared with without and aqueous or alkaline keratin dialysate, although this was only evaluated visually. Further confirmation by gene-level analysis would be beneficial to confirm the expression of proteins seen by IF staining.

HuFibs, which proliferated less than HCKs, also exhibited only low collagen I and αSMA signals throughout the 28-day period (Figure 4B). A modest increase in both markers occurred over time, with αSMA showing a slightly stronger response in HAMAKerW. Surprisingly, collagen I remained very low compared with its high output in 2D sheets [35], indicating that the culture conditions, such as the 3D HAMA hydrogel environment, which were not natural, might have suppressed their collagen production and overall function besides being viable. In addition, these results may also reflect poor cell–matrix interactions or a lack of bioactivity necessary to support the HuFib phenotype in this specific 3D environment. Furthermore, mechanotransduction could also play a role, as all hydrogel formulations demonstrated similar soft hydrogels, which could have contributed to the quiescent state. A stiffer hydrogel contributes more to a change towards myofibroblasts [52]. To further prove this, more studies on quiescent markers, such as keratocan or aldehyde dehydrogenase class 3, should be done with either IF or gene analysis [53].

## 4. Conclusions

This study evaluated the suitability of keratins as an alternative biomaterial to established collagen in a methacrylated hyaluronic acid hydrogel for corneal bioprinting application. These data demonstrated that keratins or collagen, when added to HAMA, exhibit comparable biomechanical properties and support high cell viability for corneal keratocytes and fibroblasts. Small variations could be seen in optical transparency for aqueous keratin dialysate, and a higher cell number could be observed in the collagen HAMA bioink. Nevertheless, the minor differences do not compromise the overall suitability of keratins for corneal application. These data suggest that HAMA itself has the most dominant effect on cellular behavior. Despite the differentiation medium used for the corneal fibroblasts, no proliferation nor high expression of collagen could be seen, resulting in a seemingly quiescent state of the fibroblasts in this new environment. Further studies are needed to understand how HAMA or this bioink environment affects corneal fibroblasts. Based on this result and considering the limited use of keratins in bioprinting, to our knowledge, we propose that keratins are a valuable addition to tissue engineering. Furthermore, keratins could be a promising biomaterial not only for corneal bioprinting but also in other areas of bioprinting, such as skin bioprinting. 

## Data Availability

All data or related information supporting the conclusions of this study are included in this article or in the supplementary data.

## Supplementary Materials

The following supporting information can be downloaded at https://www.mdpi.com/article/10.3390/bioengineering13060670/s1, Figure S1: Cell viability of HCKs over the course of four weeks in HAMACol, HAMAKerW and HAMAKerAlk; Figure S2: Cell viability of HuFibs over the course of four weeks in HAMACol, HAMAKerW and HAMAKerAlk; Figure S3: Relative fluorescence index for (A) HCK and (B) HuFib of HAMA, HAMACol, HAMAKerW and HAMAKerAlk bioink (*n* = 3); Figure S4: Indirect immunofluorescence of HCKs on day 7 and day 28 of HAMACol, HAMAKerW, and HAMAKerAlk; Figure S5: Indirect immunofluorescence of HuFibs on day 7 and day 28 of HAMAKerW and HAMAKerAlk and day 14 for HAMACol; Figure S6: Representative stress–strain curve of tensile testing of bioink formulations.

## Abbreviations

The following abbreviations are used in this manuscript:

PEGDA	Poly (ethylene-glycol) diacrylate
HA	Hyaluronic acid
HAMA	Methacrylated hyaluronic acid
RGD	Arginine–glycine–aspartic acid
LDV	Leucine–aspartic acid–valine
αSMA	α-smooth muscle actin
SDS	Sodium dodecyl sulfate
FBS	Fetal bovine serum
BSA	Bovine serum albumin
KGM	Keratinocyte growth medium
PBS	Phosphate-buffered solution
LAP	Lithium phenyl (2,4,6-trimethylbenzoylphosphinat)
HCKs	Human corneal keratocytes
HuFibs	Human corneal fibroblasts
HAMAKerW	HAMA with aqueous keratin dialysate
HAMAKerAlk	HAMA with alkaline keratin dialysate
HAMACol	HAMA with collagen type I
IF	Indirect immunofluorescence
UTS	Ultimate tensile strength
