# Supplementary material for: Keratin-Laden Bioink for Corneal Stroma Bioprinting
Source: Bioengineering (Basel). 2026 Jun 9;13(6):670. doi: 10.3390/bioengineering13060670 (PMC13296209; doi:10.3390/bioengineering13060670)
Supplement: Supplementary file 1 [file bioengineering-13-00670-s001.zip › bioengineering-4269586-supplementary.pdf]

Supplementary Information

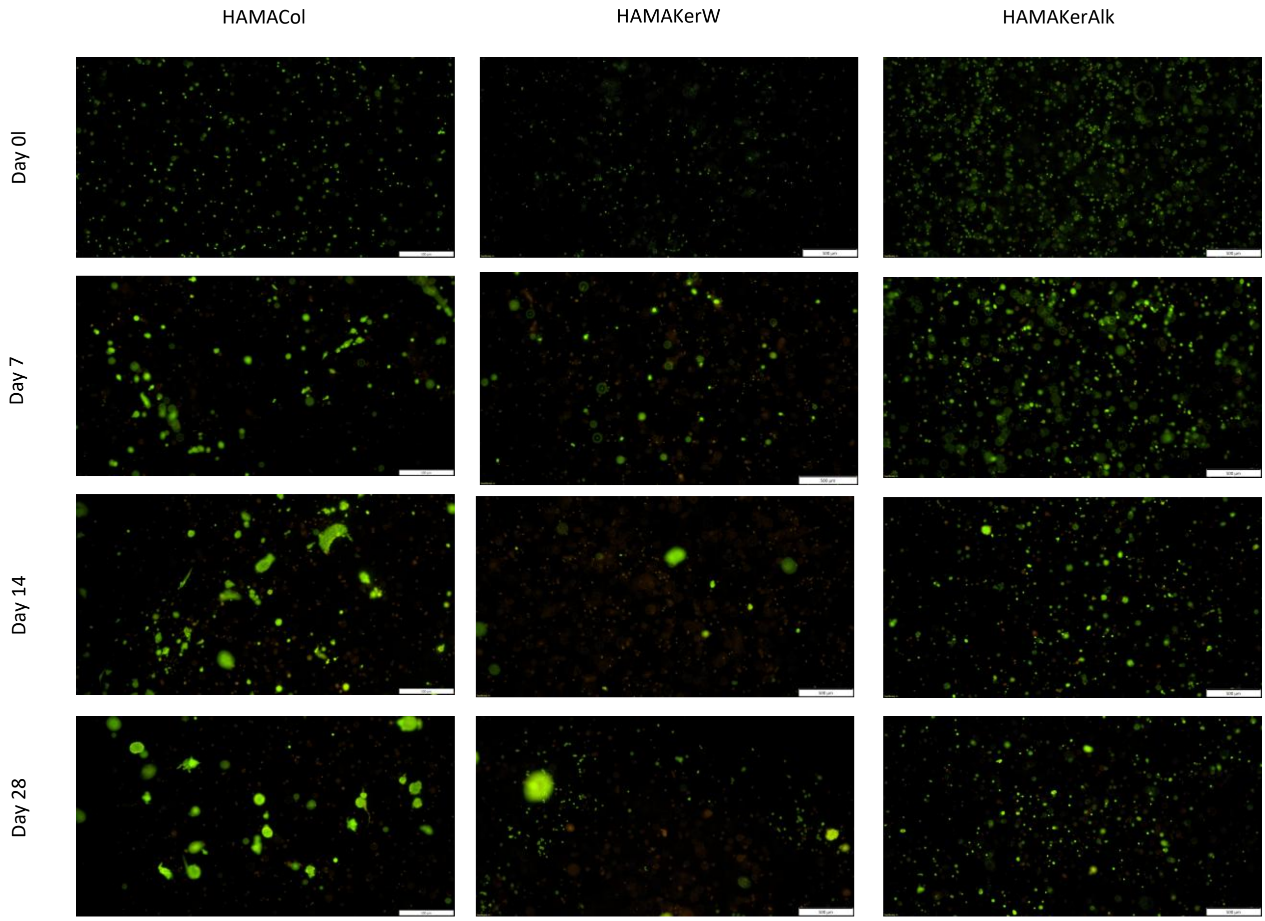

Figure S1: Cell viability of HCKs over the course of four weeks in HAMACol, HAMAKerW and HAMAKerAlk

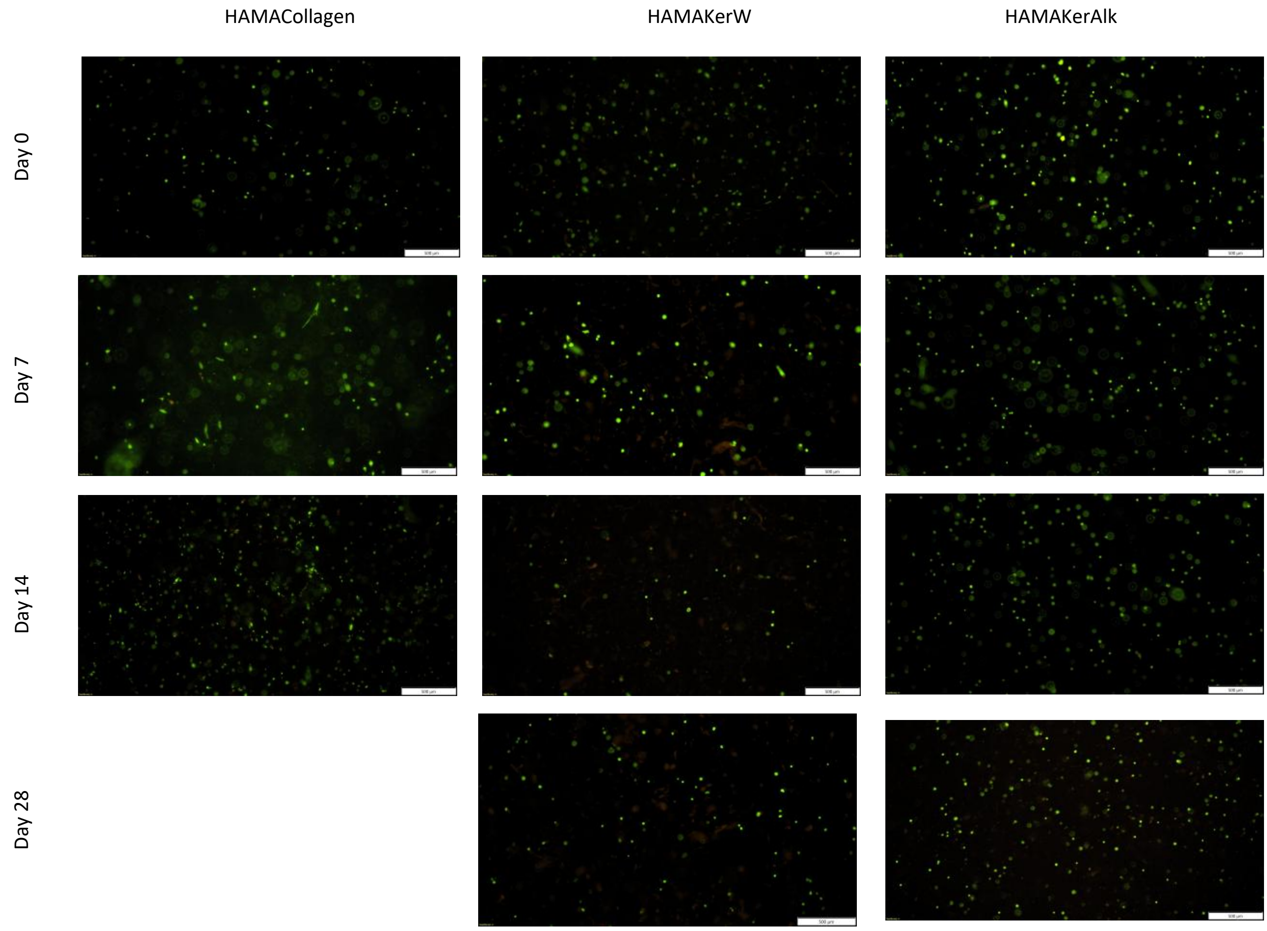

Figure S2: Cell viability of HuFibs over the course of four weeks in HAMACol, HAMAKerW and HAMAKerAlk

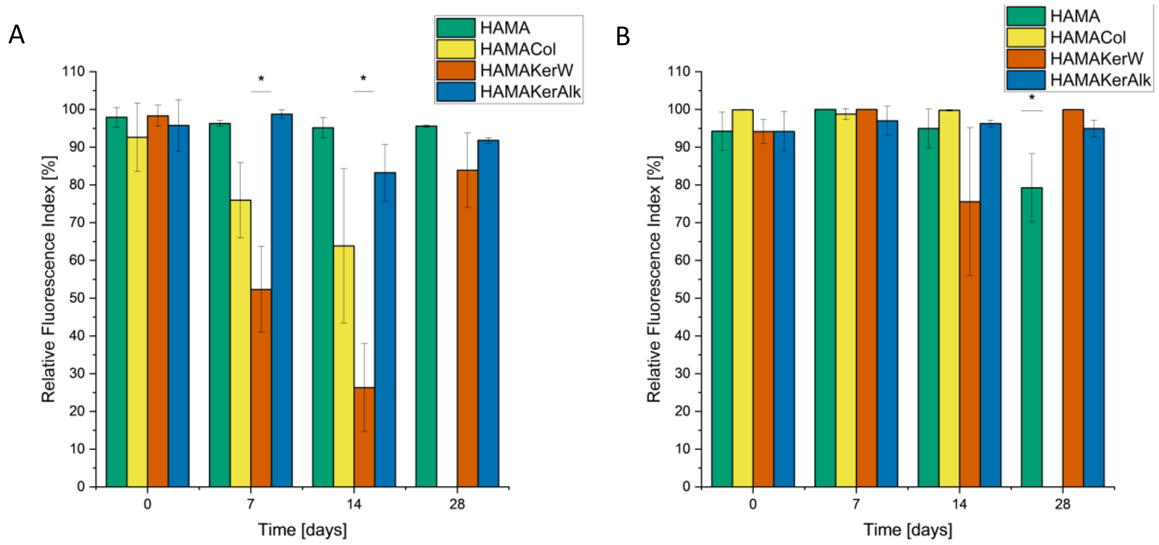

Figure S3: Relative fluorescence index for (A) HCK and (B) HuFib of HAMA, HAMACol, HAMAKerW and HAMAKerAlk bioink. (n=3)

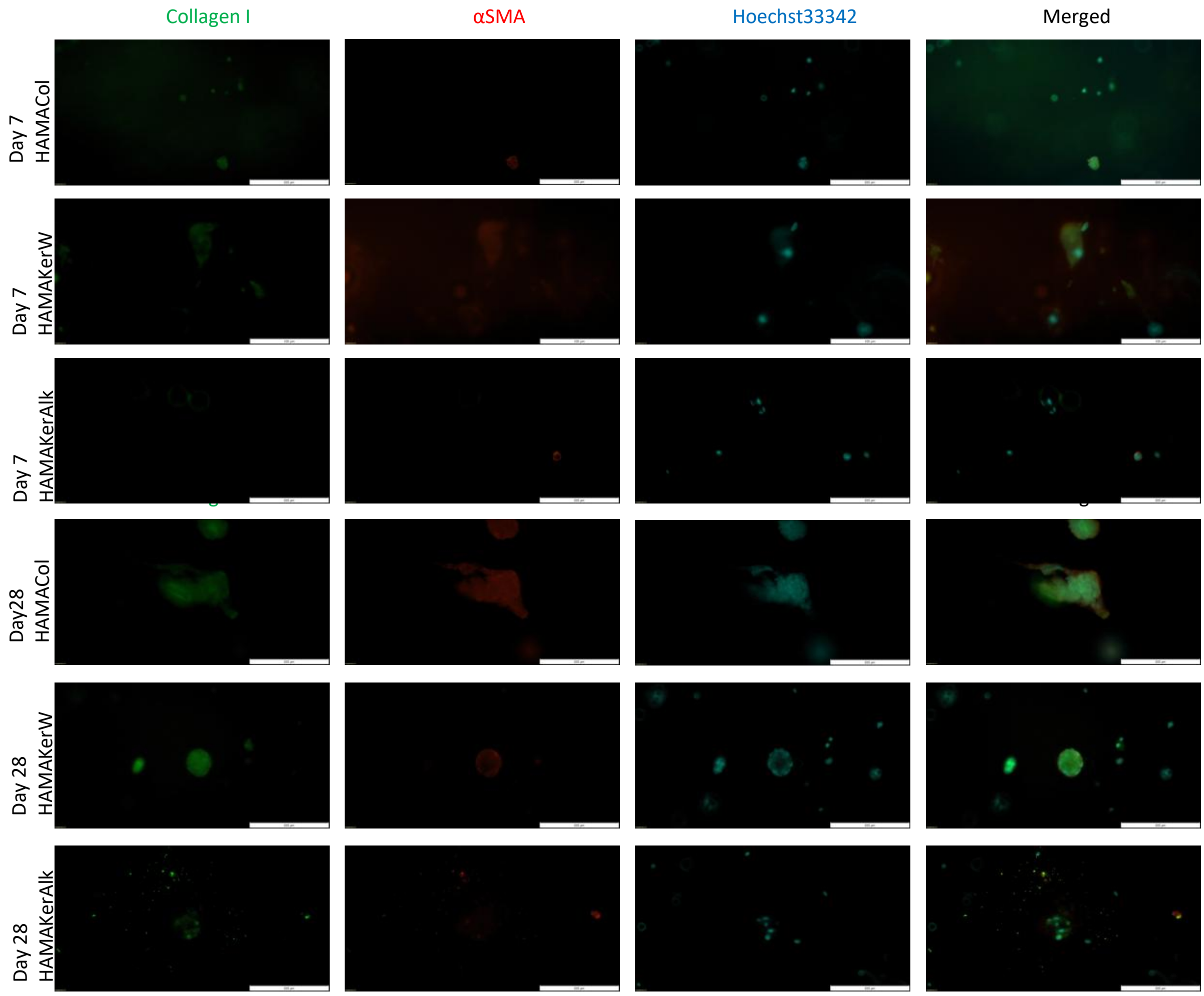

Figure S4: Indirect immunofluorescence of HCKs on day 7 and day 28 of HAMACol, HAMAKerW, HAMAKerAlk

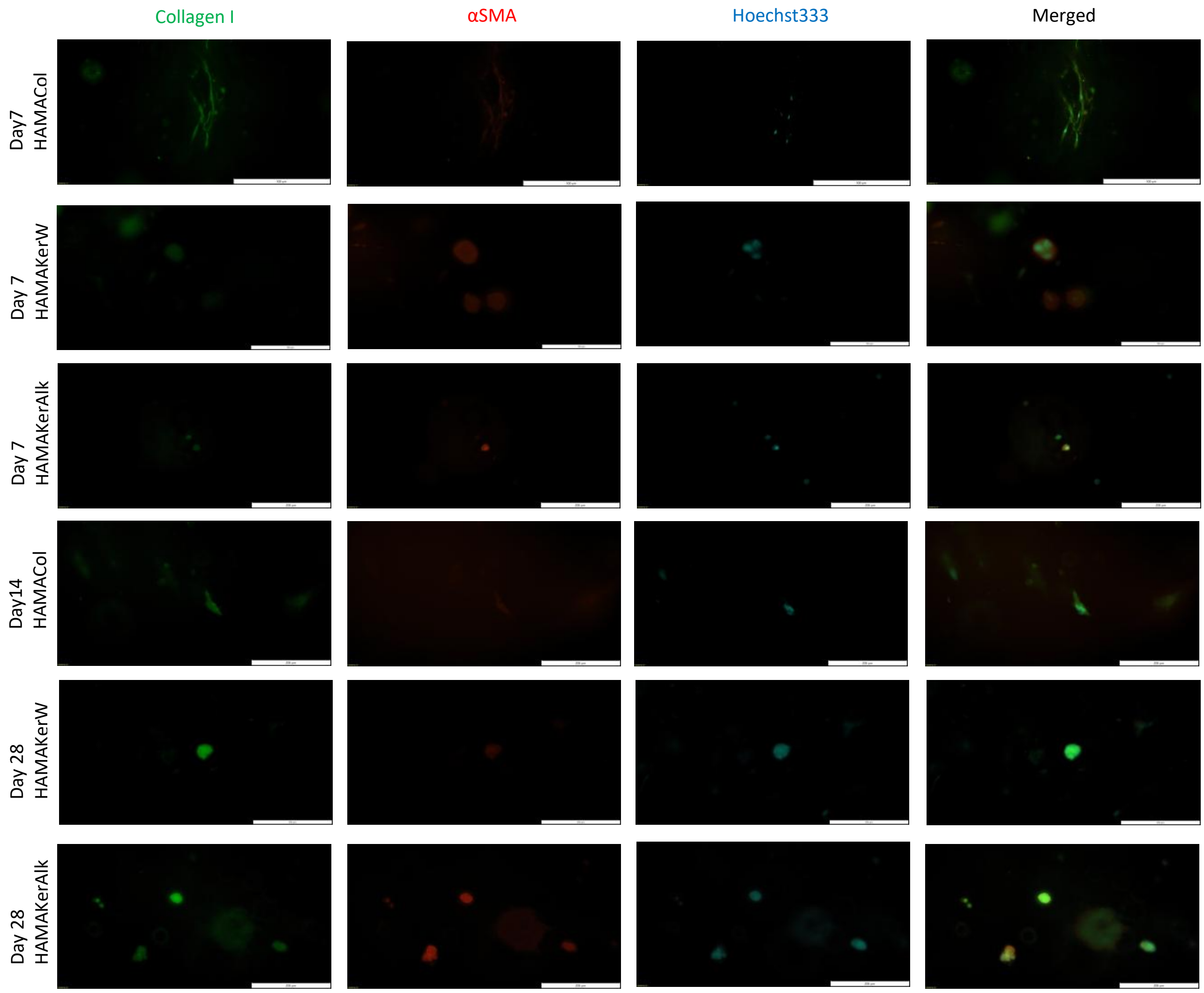

Figure S5: Indirect immunofluorescence of HuFibs on day 7 and day 28 of HAMAKerW, HAMAKerAlk and day 14 for HAMACol

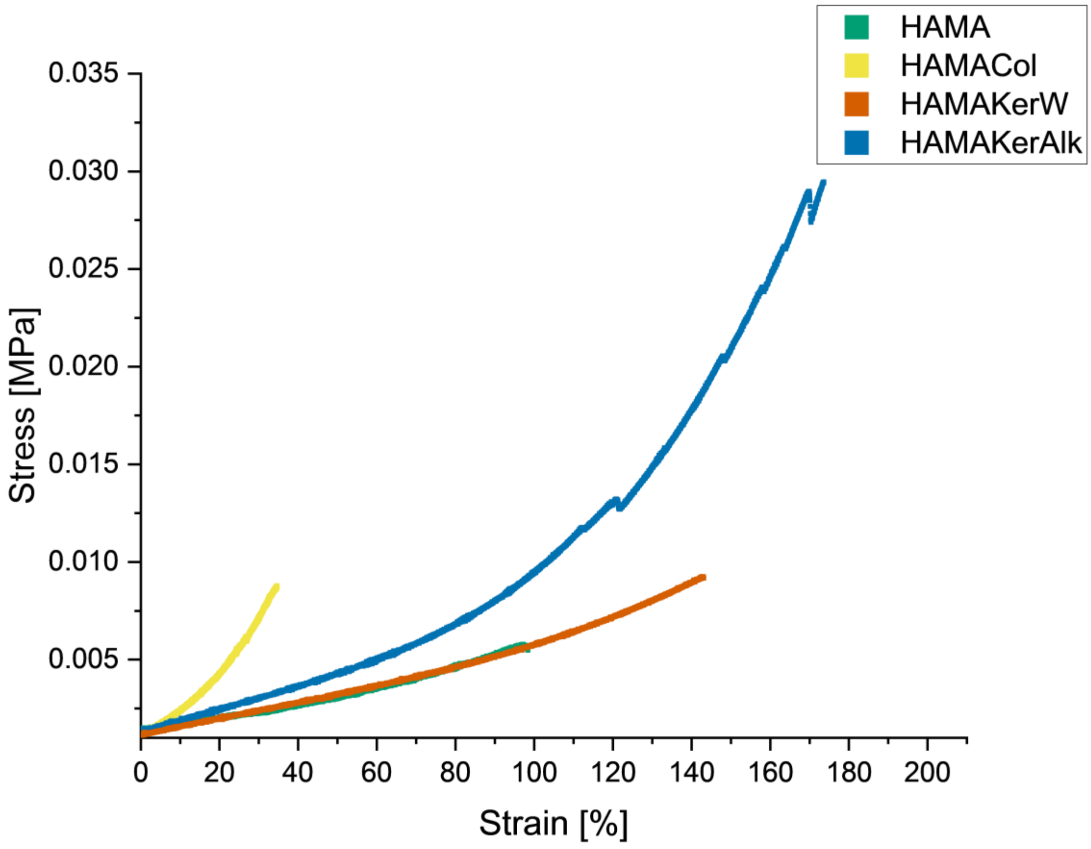

Figure S6: Representative Stress-Strain curve of Tensile Testing of bioink formulations
